# Concordance of *ompA* types in children re-infected with ocular *Chlamydia trachomatis* following mass azithromycin treatment for trachoma

**DOI:** 10.1371/journal.pntd.0010237

**Published:** 2022-03-28

**Authors:** Arman Mosenia, Stephanie A. Chin, Wondu Alemayehu, Muluken Melese, Takele Lakew, Zhaoxia Zhou, Thuy Doan, Vicky Cevallos, Thomas M. Lietman, Jeremy D. Keenan

**Affiliations:** 1 Francis I. Proctor Foundation, University of California, San Francisco, California, United States of America; 2 School of Medicine, University of California, San Francisco, California, United States of America; 3 Orbis International, New York, New York, United States of America; 4 Department of Ophthalmology, University of California, San Francisco, California, United States of America; 5 Department of Epidemiology & Biostatistics, University of California, San Francisco, California, United States of America; RTI International, UNITED REPUBLIC OF TANZANIA

## Abstract

**Background:**

The chlamydial major outer membrane protein, encoded by the *ompA* gene, is a primary target for chlamydial vaccine research. However, human studies of *ompA*-specific immunity are limited, and prior studies have been limited in differentiating re-infection from persistent infection. The purpose of this study was to assess whether children living in trachoma-endemic communities with re-infections of ocular chlamydia were more likely to be infected with a different or similar genovar.

**Methodology and findings:**

The study included 21 communities from a trachoma-hyperendemic area of Ethiopia that had been treated with a mass azithromycin distribution for trachoma. Conjunctival swabbing was offered to all children younger than 5 years of age at baseline (i.e., pre-treatment), and then at follow-up visits 2 and 6 months later. Swabs were subjected to polymerase chain reaction (PCR) to detect *C*. *trachomatis*. A random sample of 359 PCR-positive swabs, stratified by study visit and study community, was chosen for *ompA* sequencing. In addition, *ompA* sequencing was performed on all swabs of 24 children who experienced chlamydial re-infection (i.e., positive chlamydial test before treatment, negative test 2 months following mass distribution of azithromycin, and again a positive test 6 months post-treatment). *ompA* sequencing was successful for 351 of 359 swabs of the random sample and 44 of 48 swabs of the re-infection sample. In the random sample, *ompA* types clustered within households more than would be expected by chance. Among the 21 re-infected children with complete *ompA* data, 14 had the same *ompA* type before and after treatment.

**Conclusion:**

The high frequency of *ompA* concordance suggests incomplete genovar-specific protective immunity and the need for multiple antigens as vaccine targets.

## Author summary

Trachoma is the world’s leading infectious cause of blindness. Periodic mass azithromycin distributions have not resulted in elimination. Re-infection is thought to be an important barrier to elimination, but studies of serovar- or genovar-specific immunity and re-infection with ocular *Chlamydia trachomatis* have been limited. The present study investigated chlamydial genotypes in children who were infected with ocular chlamydia before azithromycin treatment and were confirmed to have cleared the infection 2 months later, but then were again infected at 6 months. The majority of re-infections were caused by the same *ompA* strain that was carried pre-treatment, which suggests poor protective immunity to chlamydia. The *ompA* types clustered by household, suggesting members of the same household as a source of re-infection. These findings could inform future mass treatment strategies and vaccine development.

## Introduction

The major outer membrane protein (MOMP), coded by the *ompA* gene, is an immunodominant antigen of human *Chlamydia trachomatis* infections, and a primary target for vaccine development. [1] Chlamydial serovar classification is based on the MOMP, with serovars A-C associated with trachoma, D-K with urogenital infections, and L1-L3 with lymphogranuloma venereum. Animal studies suggest that natural protective immunity mediated through MOMP may be serovar-specific, since experimental re-infection with the same serovar causes less clinical inflammation than the primary infection, whereas re-infection with a different serovar produces a similar degree of inflammation as the first infection. [2] Studies of genital infections in humans have found some supportive evidence of serovar-specific immunity, since re-infections have usually been found to be caused by different serovars than the initial infection. [3,4] However, existing studies are limited by relatively low sample sizes, challenges in differentiating re-infection from persistent infection, and variable study timepoints.

A cluster-randomized trial of mass azithromycin distribution for trachoma provided an opportunity to further assess the genovar-specificity of natural protective immunity in *C*. *trachomatis*. [5,6] The trial was conducted in an area with hyperendemic trachoma, and thus re-infection after antibiotic treatment was relatively frequent. Participants were likely to be exposed to the same strains of ocular chlamydia before and after treatment given the geospatial clustering observed in trachoma. [7] Conjunctival swabs were collected on all pre-school children at standardized timepoints before and after azithromycin treatment, regardless of symptoms. A sample of swabs underwent sequencing for the *ompA* gene, which codes for the immunodominant antigen of *C*. *trachomatis*. We assessed the likelihood that children with an infection at baseline who were subsequently re-infected had the same *ompA* type during both infections. We hypothesized a lack of genotype-specific protective immunity, which would result in a relatively high proportion of re-infections with the same *ompA* type as the original infection.

## Methods

### Ethics statement

Ethical approval was obtained from the University of California, San Francisco and the National Ethical Clearance Committee of the Ethiopian Science and Technology Commission. Due to high illiteracy in the study area, guardians provided verbal informed consent for all study activities.

### Study design

**Fig 1:**
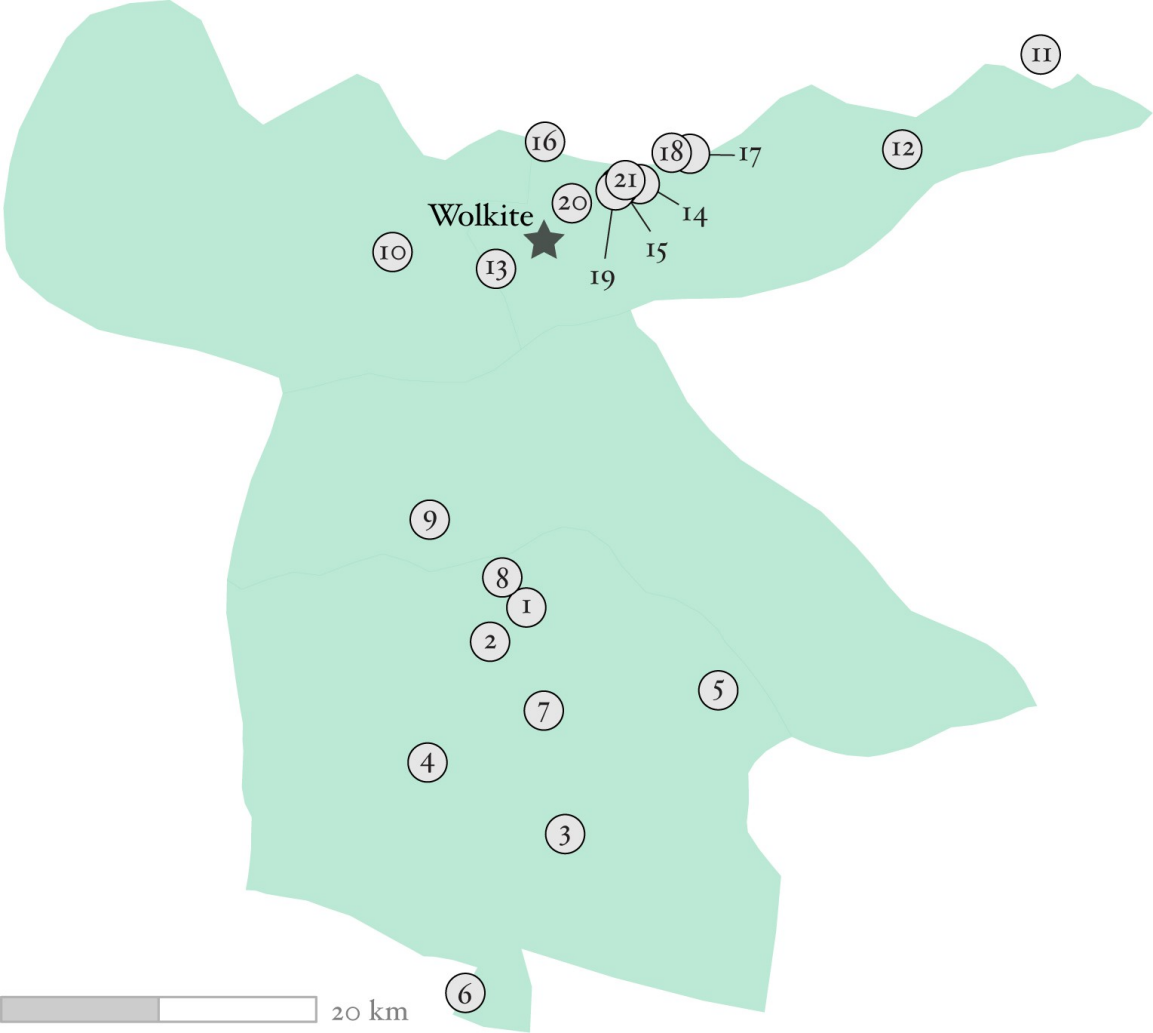
Map of the 21 study communities. Communities were part of a randomized trial set in the Gurage Zone of Ethiopia. The star depicts the capital of the zone, Wolkite. Map boundaries were taken from GADM version 4.0 (https://gadm.org).

This is a secondary observational analysis of data that was collected for a cluster-randomized trial set in the Gurage zone of Ethiopia from 2003–2006. [5,6] In the trial, 40 communities had a baseline enumerative census and monitoring visit, and then received mass azithromycin treatment of their entire population (20mg/kg for children, 1 g for adults) and no further treatments until after the month 6 monitoring visit. All children aged 1–5 years were offered conjunctival swabbing for detection of *C*. *trachomatis* with the AMPLICOR polymerase chain reaction (PCR) assay (Roche Diagnostics USA, Indianapolis, Indiana) at baseline and then 2 and 6 months following antibiotic treatment. The 21 communities with the highest prevalence of infection at month 2 were selected for the present study (Fig 1). All 21 communities had received mass azithromycin distribution at baseline, with a mean antibiotic coverage of 89% (95% confidence interval [CI] 86–92%) in the 20 communities with available coverage data. Coverage data was lost for 1 community known to have been treated with mass azithromycin. All swabs with positive chlamydial PCR results from month 2 were selected for *ompA* sequencing, as was a random sample of the same number of swabs per community from the baseline and month 6 visits (i.e., a stratified random sample). The *ompA* sequences from this random sample have been reported previously. [8,9] In addition, swabs from all children with a positive *C*. *trachomatis* result at baseline, negative result at month 2, and positive result at month 6 were selected for *ompA* sequencing. Methods for polymerase chain reaction amplification and sequencing of the *ompA* gene region have been reported previously. [8] A single nucleotide polymorphism was considered a different *ompA* genotype. *ompA* types were labeled with genovar (A or B) and an arbitrary number (i.e., A1 to A11 and B1 to B4).

### Statistical analyses

All households in which more than 1 child contributed an *ompA* result were included in a permutational multivariate analysis of variance (PERMANOVA) test to assess whether dissimilarity in *ompA* types was greater between households than within households (child-level analysis; Euclidean distance matrix of binary *ompA* indicator variables; dissimilarity partitioned over the nested hierarchical classes of community and household; 10,000 repetitions). Children with a positive *C*. *trachomatis* result at baseline, negative result at 2 months, and positive result at 6 months were considered to have cleared infection after antibiotic treatment and been subsequently re-infected. These children were included in a permutation test to assess whether the observed within-person concordance of the baseline and month 6 *ompA* types was higher than would be expected by chance. A significance level of 0.025 was set for each analysis. Analyses and plots were done in R version 4 (R for Statistical Computing, Vienna Austria).

## Results

**Fig 2:**
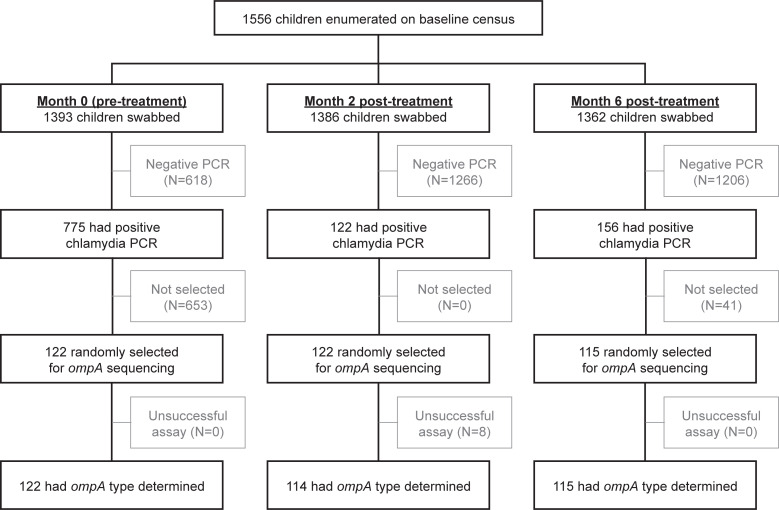
Flow diagram. Participant flow is shown separately for baseline (i.e., pre-treatment), month 2, and month 6.

A total of 1556 children aged 1–5 years were enumerated in the baseline census from the 21 study communities. Conjunctival swabs were collected from 1393 children at baseline, 1386 children at month 2, and 1362 children at month 6 (Fig 2). The mean community-level prevalence of *C*. *trachomatis* among 1–5 year-olds was 56% (95%CI 49–64%) before treatment, 9% (95%CI 7–11%) 2 months after the antibiotic distribution, and 12% (95%CI 9–15%) 6 months after antibiotics. [8]

**Fig 3:**
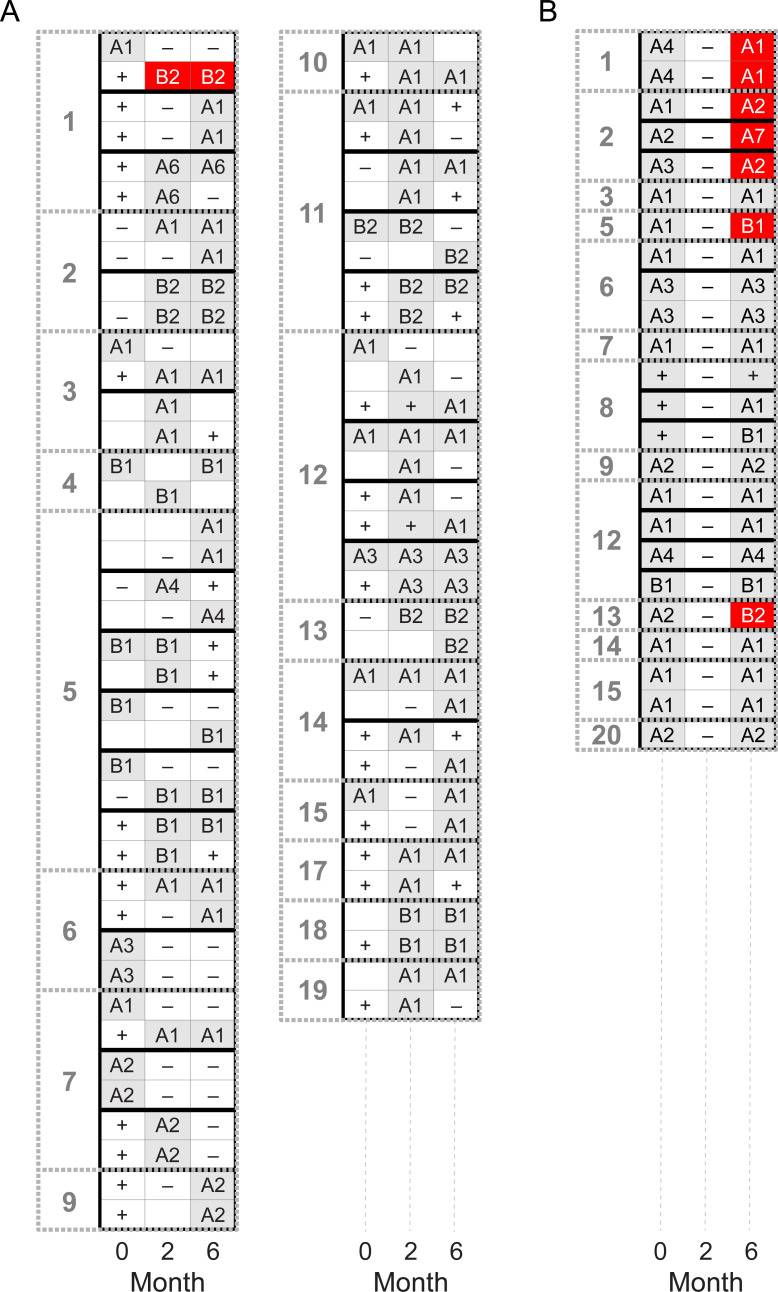
Concordance of *ompA* type in 21 Ethiopian communities. Panel A shows all households in the random sample that had multiple children with *ompA* results. Each row of boxes represents a unique child’s *ompA* results over time, from before the mass azithromycin distribution (i.e., month 0) to 6 months post-treatment. Blank spaces represent visits with no specimen collected. Shaded boxes represent those swabs selected for the study, and hence those eligible for *ompA* sequencing. Shaded boxes are labeled with the sequenced *ompA* type using the arbitrary naming conventions of this study (i.e., A1, A2, B1, etc.) or with “+” if the *ompA* type could not be determined. Unshaded boxes are labeled according to the polymerase chain reaction (PCR) results for *C*. *trachomatis* as positive (“+”) or negative (“-“). Households are delineated with thick black lines and communities with grey dashed lines. Panel B shows all children experiencing *Chlamydia trachomatis* re-infection after mass azithromycin treatment (i.e., positive test results at baseline, negative results at month 2, and positive results again at month 6), regardless of whether they were selected for the random sample. Discordant *ompA* types are shown in red.

The stratified random sample consisted of 122 swabs from baseline, 122 swabs from month 2, and 115 swabs from month 6, with equal numbers of swabs from each community at each study timepoint except for 4 communities with fewer PCR-positive results at month 6. Of 359 swabs selected as part of the random sample across the 3 timepoints, *ompA* sequencing was successful for 351 (98%). The nucleotide sequences of each of these *ompA* types have been reported elsewhere; the present study follows the same numbering system as the previous report. [8] A total of 15 different *ompA* types were identified, ranging from A1 to A11 and from B1 to B4. Each *ompA* type had at least one amino acid difference relative to the other sequences. [8] The most common *ompA* types were A1, which accounted for about half of swabs (N = 64 at baseline, N = 57 at month 2, and N = 57 at month 6), and B1, which accounted for almost 15% of swabs (N = 18 at baseline, N = 16 at month 2, and N = 16 at month 6). Data on *ompA* type were contributed by a total of 274 children (mean age 3.2 years; 48% female) from 237 households, with 36 households contributing more than 1 child. Of these 36 households, 35 contained only a single *ompA* type across all children and/or timepoints (Fig 3A). The *ompA* types of a given community were statistically significantly more similar within the same household than between different households (PERMANOVA *P*<0.001).

A total of 24 children had a swab positive for *C*. *trachomatis* at baseline, negative at 2 months, and then positive again at 6 months, and 21 of these children had successful *ompA* sequencing results at both the baseline and 6-month visits. Of these 21 children, 14 (67%, 95%CI 43–85%) had the same strain at both timepoints, which was significantly more than would be expected by chance (permutation *P*<0.001; Fig 3B). In 3 households, two siblings experienced re-infections. In each case, pairs of siblings shared the same *ompA* type at each timepoint, with two of the pairs having the same *ompA* type at baseline and 6 months, whereas the other pair was re-infected with a different *ompA* type.

## Discussion

It is well established that children living in areas with hyperendemic trachoma are likely to experience repeated infections with ocular chlamydia, but the serovars or genovars of re-infections have not been well described. [6] This study found that two-thirds of children re-infected with ocular strains of *C*. *trachomatis* after mass azithromycin treatment were re-infected with the same *ompA* type. The *ompA* types of children living in the same household were more similar than the *ompA* types of children living in different households of the same community, which is compatible with the theory that household members or nearby neighbors are the most likely sources of re-infection. These findings are consistent with a smaller study of Egyptian children with trachoma that found clustering of *ompA* type at the household-level (N = 34 households) and re-infection with the same *ompA* type at the individual level (N = 3 children) [10]. The results confirm that natural protective immunity to chlamydia is incomplete and suggest that chlamydia vaccines relying on MOMP alone may be less effective than those relying on multiple antigenic epitopes.

Studies of sexually transmitted chlamydia infections have also documented re-infections with the same *ompA* type, but at lower proportions than observed in the present study. For example, a prospective study of sex workers in Kenya found that 15 of 40 (38%) women with two positive tests for *C*. *trachomatis* more than 4 weeks apart had the same *ompA* type at both tests. [4] In a retrospective study of 74 patients from an Indiana clinic with two positive chlamydia tests, 88% of whom had an intervening negative chlamydia culture, 25 (34%) had the same *ompA* type at both visits. [11] A prospective study of 35 patients from a clinic in Alabama with positive tests for *C*. *trachomatis* before and 6 months after azithromycin therapy found that 7 (20%) had the same *ompA* type at both timepoints. [3] However, the generalizability of these studies is unclear. Two studies did not document a negative test between the two positive tests, making it difficult to differentiate re-infection from persistent infection. [3,4] Two studies assessed for re-infections at variable timepoints [4,11]. Most re-infections from the Kenya study occurred in women with HIV, which may have resulted in altered natural immunity. Moreover, people re-infected with genital *C*. *trachomatis* may be exposed to more diverse set of serovars and genovars than children with trachoma, who are likely exposed to the same *ompA* types in circulation in the community. [12]

The study has several strengths, including its population-based sampling, standardized timepoints, strict definition of re-infection (i.e., requirement of a negative post-treatment chlamydial test) and setting in a place with a high chance of being re-exposed to the same *ompA* type after antibiotic treatment. The study’s biggest limitation is its focus on a single antigenic epitope, the MOMP. While MOMP is probably the most important immunodominant epitope and the target of much vaccine research, more extensive sequencing could have provided information about other immunogenic epitopes.

In summary, two-thirds of children who successfully cleared ocular *C*. *trachomatis* infection after mass azithromycin treatment but were subsequently re-infected frequently had the same *ompA* type during the original infection and the re-infection. *ompA* types clustered by household over the duration of the study, suggesting fellow household members could be potential sources of re-infection. Chlamydia vaccines targeting only the MOMP may be less likely to confer protective immunity than vaccines containing multiple antigenic epitopes.

## Supporting information

S1 Data *ompA* types in random sample. A stratified random sample of children aged 1–5 years from 21 communities in the Gurage Zone, Ethiopia who had a positive polymerase chain reaction (PCR) test for *Chlamydia trachomatis* underwent sequencing for the ompA gene. Separate stratified random samples were selected before mass azithromycin treatment, 2 months after treatment, and 6 months after treatment.
(CSV)

S2 Data *ompA* types in re-infection sample. All children aged 1–5 years from 21 communities in the Gurage Zone, Ethiopia who had polymerase chain reaction (PCR) evidence of ocular *Chlamydia trachomatis* infection before mass azithromycin treatment, then a negative PCR test 2 months after treatment, and then again a positive PCR test 6 months after treatment underwent *ompA* sequencing.
(CSV)
